# Measuring the impact of the Affordable Care Act Medicaid expansion on access to primary care using an interrupted time series approach

**DOI:** 10.1186/s12961-021-00730-0

**Published:** 2021-05-06

**Authors:** Elizabeth A. Brown, Brandi M. White, Walter J. Jones, Mulugeta Gebregziabher, Kit N. Simpson

**Affiliations:** 1 Department of Clinical Sciences, College of Health Professions, Medical University of South Carolina, 151-B Rutledge Avenue, MSC 962, Charleston, SC 29425 USA; 2 Division of Health Sciences, Education, and Research, College of Health Sciences, University of Kentucky, Room 209C Wethington Building, 900 South Limestone Street, Lexington, KY 40536-0200 USA; 3 Department of Healthcare Leadership and Management, College of Health Professions, Medical University of South Carolina, 151-B Rutledge Ave, MSC 962, Charleston, SC 29425 USA; 4 Department of Public Health Sciences, College of Medicine, Medical University of South Carolina, 135 Cannon Street, Charleston, SC 29425 USA

**Keywords:** Access, Primary care, Medicaid, Patient Protection and Affordable Care Act, Health policy, Interrupted time series analysis

## Abstract

**Background:**

The Patient Protection and Affordable Care Act of 2010, commonly referred to as the Affordable Care Act (ACA), was created to increase access to primary care, improve quality of care, and decrease healthcare costs. A key provision in the law that mandated expansion of state Medicaid programme changed when states were given the option to voluntarily expand Medicaid. Our study sought to measure the impact of ACA Medicaid expansion on preventable hospitalization (PH) rates, a measure of access to primary care.

**Methods:**

We performed an interrupted time series analysis of quarterly hospitalization rates across eight states from 2012 to 2015. Segmented regression analysis was utilized to determine the impact of policy reform on PH rates.

**Results:**

The Affordable Care Act’s Medicaid expansion led to decreased rates of PH (improved access to care); however, the finding was not significant (coefficient estimate: −0.0059, CI −0.0225, 0.0107, *p* = 0.4856). Healthcare system characteristics, such as Medicaid spending per enrollee and Medicaid income eligibility, were associated with a significant decrease in rates of PH (improved access to care). However, the Medicaid-to-Medicare fee index (physician reimbursement) and states with a Democratic state legislature had a significant increase in rates of PH (poor access to care).

**Conclusion:**

Health policy reform and healthcare delivery characteristics impact access to care. Researchers should continue evaluating such policy changes across more states over longer periods of time. Researchers should translate these findings into cost analysis for state policy-makers to make better-informed decisions for their constituents.

**Contribution to knowledge:**

Ambulatory care-sensitive conditions are a feasible method for evaluating policy and measuring access to primary care. Policy alone cannot improve access to care. Other factors (trust, communication, policy-makers’ motivations and objectives, etc.) must be addressed to improve access.

**Supplementary Information:**

The online version contains supplementary material available at 10.1186/s12961-021-00730-0.

## Background

One of the most influential factors impacting access to primary care in the United States is the implementation of federal healthcare legislation, policies, and programmes. Although healthcare legislation aims to create equity in healthcare, many Americans still lack access to quality medical care for a host of reasons, including their race/ethnicity [1] or socioeconomic factors like income [2] and employment [3]. In an effort to increase access to primary care, improve quality of care, and decrease unnecessary costs in healthcare, legislators created the Patient Protection and Affordable Care Act (PPACA) of 2010, commonly referred to as the Affordable Care Act (ACA) or “Obamacare”. The ACA’s fundamental provisions included mandates such as (a) expanding Medicaid eligibility to 138% of the federal poverty level (FPL) in all states, (b) modifying Medicaid eligibility, allowing individuals with low incomes and without dependents to be eligible for Medicaid, (c) requiring individuals to secure health insurance or face a tax penalty, and (d) allowing children to stay on their parents’ health insurance until their 26th birthday [4].

### Conceptual framework

**Fig. 1:**
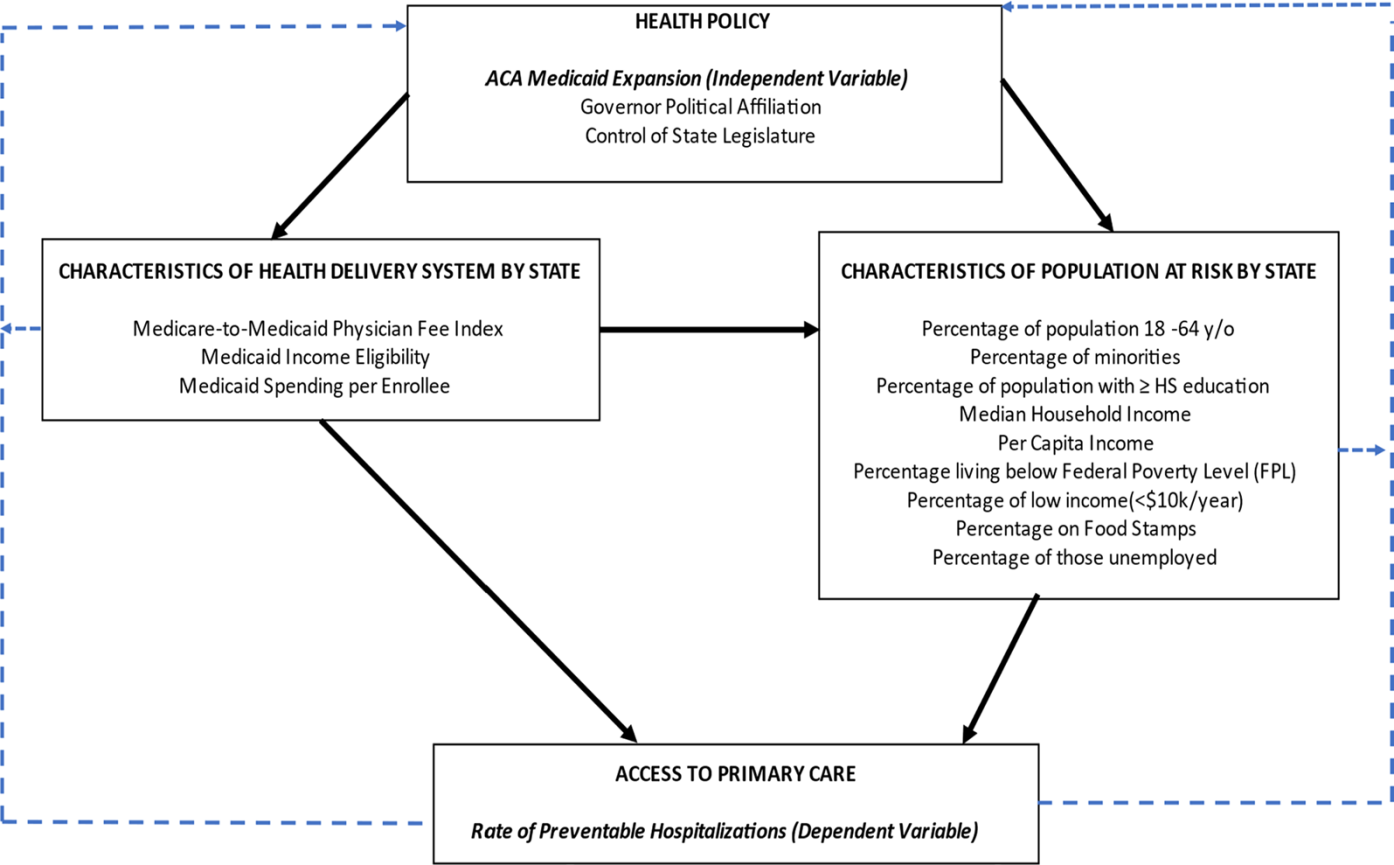
Conceptual framework for access to primary care, Adapted from Aday and Andersen’s framework for the study of access

Access to primary care is a versatile concept, incorporating an array of variables that influence the ability to receive and maintain care. According to the theoretical model—framework for the study of access—when examining access to care, the following personal and environmental factors can significantly influence access to care: health policy, delivery of care, individual characteristics, utilization of care, and patient satisfaction [5]. The framework identifies and discusses several variables that may either impede or promote access to care. The authors employ several constructs in Aday and Andersen’s framework for the study of access, including healthcare policy, healthcare delivery, and sociodemographic characteristics, to guide the research study and select appropriate variables for analytic purposes (Fig. 1).

Regarding healthcare policy, former President Barack Obama and supporters aspired to increase access to primary care with the ACA, one of the most significant healthcare reforms in over 40 years. However, in June 2012, the Supreme Court “applied a theory of coercion” [6], in *National Federation of Independent Business v. Sebelius*, striking down the federal government’s mandate for state Medicaid expansion without financial repercussions to states, thus giving states the option to voluntarily expand Medicaid or opt out of Medicaid expansion without any monetary penalties from the federal government [6]. The court’s ruling modified the ACA and potentially had a detrimental impact on access to care, hospitals, and health outcomes for millions of Americans with low incomes, especially for people living in states that chose not to expand their Medicaid programme [7].

States with Republican governors were more likely to opt out of Medicaid expansion, while states with Democratic governors were more likely to choose to expand Medicaid [8]. Many governors in opt-out states cited constrained state budgets and uncertainty regarding the federal government’s ongoing financial support towards Medicaid expansion; however, governors in states that expanded believed expansion would increase access by covering the uninsured population, improve health outcomes, and save tax dollars [8]. The Supreme Court ruling and policy-makers’ attitudes towards the ACA Medicaid expansion may have impacted primary care access.

### Purpose

This study compared access to primary care—measured by the rate of preventable hospitalizations (PH)—in a cohort of four states that implemented the ACA Medicaid expansion on 1 January 2014 to a cohort of four states that did not expand their Medicaid programmes.

## Methods

### Study design

The ACA’s Medicaid expansion allowed for a retrospective, quasi-experimental study using an interrupted time series (ITS) research design to evaluate the impact of the ACA Medicaid expansion on access to primary care. The ITS design allows for a change or intervention to separate time periods and compare the effect of the intervention; it is increasingly used in the evaluation of healthcare interventions such as healthcare policies and programmes [9]. ITS is a robust design for evaluating the effectiveness of a population-level intervention like Medicaid expansion that was implemented at a clearly defined point in time [10].

The intervention was the implementation of Medicaid expansion under the ACA effective 1 January 2014. We examined eight quarterly pre-intervention time points prior to implementation (1 January 2012–31 December 2013) and seven quarterly post-intervention time points after implementation (1 January 2014–30 September 2015). In 2015, fourth quarter hospital admissions (October through December) were not used due to the transition from International Classification of Diseases, Ninth Revision (ICD-9) codes to ICD-10 codes. Generally, it is suggested that researchers have 12 data points before and 12 data points after the intervention when using ITS [11]. However, researchers utilizing ITS methodology have used different number of data points before and after an intervention such as a policy change [12, 13].

### Study population

Based on hospitalization data available at the time of this project, we selected four states that expanded Medicaid on 1 January 2014 (treatment group): Arizona (AZ), Kentucky (KY), New Jersey (NJ), and New York (NY). Then, we selected four states that chose not to expand Medicaid (control group): Florida (FL), Georgia (GA), South Carolina (SC), and Wisconsin (WI). Most of these states are more likely to be generalizable to the Southeast or Northeast United States.

Inclusion criteria include the following characteristics: (a) all payers (Medicaid, Medicare, private insurance, self-pay), (b) 18–64 years old, (c) all races, and (d) all hospitalizations with an ambulatory care-sensitive (ACS) condition as the primary discharge diagnosis. South Carolina hospitalization data included those aged 20–64 years due to administrative hospital data construction. We included all community hospitals that report patient discharge data to the Agency for Health Research and Quality’s (AHRQ) Healthcare Cost and Utilization Project (HCUP) State Inpatient Databases (SID). Federal or veteran hospitals were not included in the study sample.

### Data sources

The AHRQ HCUP SID provided administrative hospital data, patient demographics, ICD-9 diagnosis codes, total charges, length of stay, and expected payment source for all hospital inpatient stays in community hospitals in each state [14].

Data from the 2010 Census and 2015 population estimates were used to compute annual population estimates (18–64 years old) from 2012 to 2015 [16]. Aggregated Census data (2011–2015) were used to report education and median household income (MHI). The 2015 American Community Survey provided the percentage of unemployed persons [17].

The National Conference of State Legislatures (NCSL) provided data on state legislature control [18]. The Henry J. Kaiser Family Foundation (KFF) provided information about the Medicaid-to-Medicare fee index and Medicaid spending per enrollee [19, 20]. KFF also reports state Medicaid income eligibility limits [21].

### Independent variables

The main independent variables were state Medicaid expansion status, post-Medicaid expansion (time), and an interaction term (state Medicaid expansion status*post-Medicaid expansion). State Medicaid expansion status was a dichotomous variable and indicated whether a state expanded its Medicaid programme as of 1 January 2014 (0 = No vs 1 = Yes). Post-Medicaid expansion (time variable) was dichotomous (0 = before 1 January 2014 vs 1 = after 1 January 2014. The interaction term was a binary variable indicating time before and after expansion, interacted with the time variable to assess change in the rate of PH after Medicaid expansion.

#### Sociodemographic model

The following state characteristics were considered independent variables in the sociodemographic model: (a) percentage of population 18–64 years old, (b) percentage of minorities, (c) percentage of people 25 years and older who had a bachelor’s degree from 2011 to 2015, (d) percentage of people unemployed, and (e) MHI from 2011 to 2015 (reported in 2015 dollars).

#### Health delivery system model

The 2014 Medicaid-to-Medicare fee index was a numeric value indicating the amount state Medicaid programmes reimbursed physicians for primary care services compared to Medicare primary care services. Medicaid income eligibility was a numeric value representing the FPL for Medicaid eligibility prior to and after 1 January 2014. Medicaid spending per adult enrollee was the dollar amount each state spent on its adult Medicaid enrollees in fiscal year 2011.

#### Health policy model

Republican state governor was a dichotomous variable (0= Democratic vs 1= Republican) and measured the period when the state had a Republican governor. The Republican state legislature was a dichotomous variable (0= Democratic vs 1= Republican) and measured the period when the Republican party had control of the state legislature.

### Dependent variable

The dependent variable was the change in the rate of PH per 10,000 persons due to ACS conditions in adults aged 18–64 years from January 2012 to September 2015 for selected states.

#### PH

PH were defined as a hospitalization with an ACS condition as the principal discharge diagnosis and were used to measure access to primary care. The use of ACS conditions is a validated method to measure access to primary care [22]. Theoretically, ACS conditions are illnesses or diagnoses for which, with proper primary care, hospitalizations can be avoided if the disease is appropriately managed in the community setting [2]. Examples of some ACS conditions included in our study were asthma, bacterial pneumonia, dehydration, hypertension, and diabetes (Additional file 1).

### Statistical analysis

We performed bivariate analysis to identify significant differences in data between the two cohorts: Medicaid expansion states (*n* = 4) and non-Medicaid expansion states (*n* = 4). Correlation between variables was examined, and moderately to highly correlated variables (*r* > 0.65) were excluded from the final analysis [23]. We performed *t*-tests for continuous data and chi-square tests for categorical data.

We used segmented regression for ITS (SR-ITS) described by Wagner and colleagues as the primary analysis approach [11]. The regression for each segment was based on a general linear model (GLM), which allowed us to estimate the level and trend changes associated with the intervention, and it also allowed us to control for baseline level and trend. We used the time series for each outcome to establish an underlying trend, which is “interrupted” by an intervention at a known point in time—1 January 2014. We assessed the effect of the intervention against the counterfactual (hypothetical scenario under which the intervention did not take place, and the trend continues unchanged given the pre-intervention period). We differentiated the pre-intervention period and the post-intervention period and the implementation period so that it could be considered separately when assessing the effect of the intervention.

A GLM was used to analyse unadjusted and adjusted models. Since we used only eight states for the analysis, we analysed separate regression models based on factors in the Aday and Andersen framework for the study of access to care [5]. The explanatory model consisted of one independent variable, Medicaid expansion status (0 vs 1), where 0 was “No” and 1 was “Yes”. Next, the base model included an interaction term (Medicaid expansion status*time), which accounted for the trend over time by quarters. The subsequent three models accounted for various characteristics, including sociodemographic factors, health delivery system factors, and health policy factors.

Main effects were considered significant at alpha < 0.05. The interaction term was considered significant at alpha < 0.15, indicating a significant change in the rate of PH due to ACS conditions after the implementation of Medicaid expansion. [24] We used SAS 9.4 software to complete all data analysis (SAS Institute Inc., Cary, NC).

An institutional review board deemed this research not to be human subject research since data used de-identified public-use data. The appropriate persons completed the HCUP Data Use Agreement (DUA) online training.

## Results

There were 2,103,114 PHs in eight states from January 2012 to September 2015. Approximately 52.1% of these PHs occurred in the cohort of states that did not expand Medicaid (Table 1). States that expanded Medicaid had a higher proportion of individuals with a bachelor’s degree (30.2% vs 27.4%, *p* = 0.0194) and reported a higher MHI ($56,240 vs $49,006, *p* = 0.0005).

**Table 1:** Demographic variables and primary care access by Affordable Care Act Medicaid expansion status, 2012–2015

Demographic variables^a^	NME^b^ states	ME^c^ states	*p*-value
Access to care (*n*, %)			
Preventable hospitalizations	1,096,631 (52.1)	1,006,483 (47.9)	0.2670
State population			
18–64 years old	6,127,025	6,191,002	0.8776
Race (%)			
Minorities	41.7	38.9	0.9457
Education (%)			
High school graduate	87.3	86.1	0.0676
Bachelor’s degree	27.4	30.2	*0.0194*
Income ($)			
MHI	49,006	56,240	*0.0005*
Per capita income	26,381	29,865	*0.0194*
Poverty (%)			
Below FPL	15.3	15.6	0.1704
Income < 10 k	7.2	7.6	0.2244
Food stamps	14.0	13.5	0.2546
Unemployment	7.0	7.2	0.2595
Healthcare delivery characteristics			
Physician fee index ($)^d^	0.60	0.58	0.8029
Medicaid $ per enrollee ($)^e^	3,336	5,1205,120	* < 0.0001*
Income eligibility, FPL (%)^f^	80.6	124.1	* < 0.0001*
Governor political affiliation			
Democratic	0 (0.0)	27 (100.0)	* < 0.0001*
Republican	62 (64.6)	34 (35.4)34 (35.4)	
Control of state legislature			
Democratic	0 (0.0)	45 (100.0)	* < 0.0001*
Republican	62 (79.5)	16 (20.5)	

Italic values are significant values

^a^*t*-test for continuous variables and chi-square test for categorical variables

^b^NME: Non-Medicaid expansion states include South Carolina, Wisconsin, Georgia, and Florida

^c^ME: Medicaid expansion states include Arizona, Kentucky, New Jersey, and New York

^d^Medicaid-to-Medicare fee index

^e^Medicaid spending per enrollee

^f^Average of pre- and post-ACA FPL Medicaid income eligibility limits for parents of dependent children (in a family of three)

**Fig. 2:**
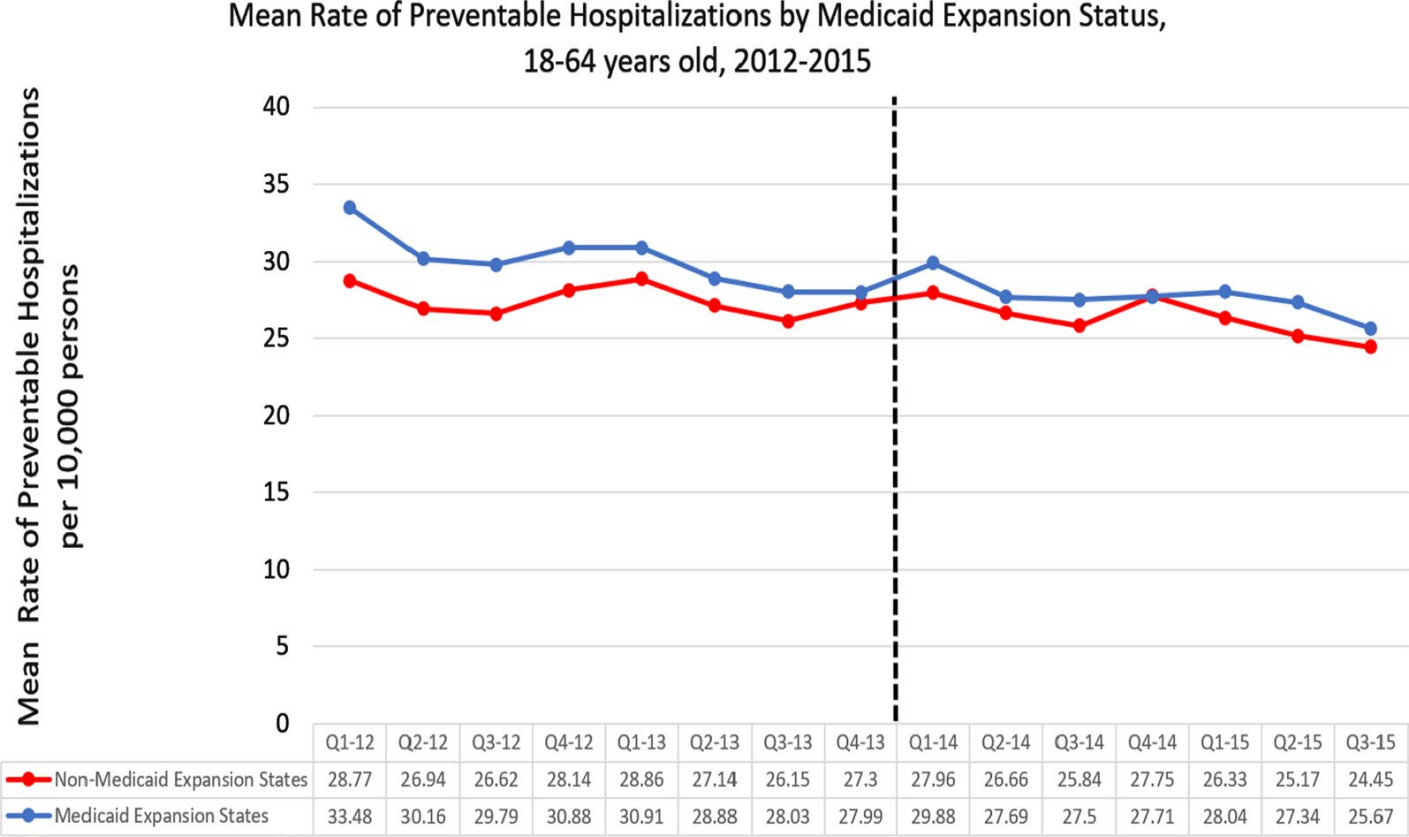
Mean rate of preventable hospitalizations per 10,000 persons by Medicaid expansion status, 2012–2015

On average, states that expanded Medicaid had a higher mean rate of PH per 10,000 persons than states that did not expand Medicaid (28.93/10,000 vs 26.94/10,000) (Fig. 2).

In 2014, states that expanded Medicaid eligibility limits had a mean 138% of the FPL compared to 60.2% of the FPL for states that did not expand (Table 2). Medicaid spending per enrollee was higher in Medicaid expansion states than in states that did not expand their Medicaid programme ($5103 vs $3342) in fiscal year 2011.

**Table 2:** Snapshot of health delivery system characteristics for selected states

States	2014 Medicaid income eligibility, %^a^	Medicaid spending per enrollee, $^b^	Medicaid-to-Medicare fee index, $^c^
Medicaid expansion			
Arizona	138	6,131	0.73
Kentucky	138	5,000	0.67
New Jersey	138	4,687	0.42
New York	138	4,596	0.44
*Mean for Medicaid expansion states*	138	5,103	0.57
Non-Medicaid expansion states			
Florida	35	2,880	0.48
Georgia	39	4,174	0.65
South Carolina	67	3,583	0.70
Wisconsin	100	2,731	0.48
*Mean for non-Medicaid expansion states*	60.2	3,342	0.58

^a^Medicaid income eligibility—2014 data, ACA FPL income eligibility limits for parents of dependent children (in a family of three)

^b^FY 2011, 1 October 2010–30 September 2011, adults only, includes full or partial Medicaid benefits

^c^Medicaid-to-Medicare fee index—2014 data

In the unadjusted model, Medicaid expansion was associated with a slight but nonsignificant increase in PH rates (coefficient estimate 0.0713, CI −0.0076, 0.1503, *p* = 0.0764) (Table 3). After accounting for trend over time, the change in PH rates in Medicaid expansion states declined; however, this finding was not significant (coefficient estimate −0.0059, CI −0.0225, 0.0107, *p* = 0.4856).

**Table 3:** Regression models for rates of preventable hospitalizations

Coefficient estimates (95% confidence intervals)		
Variables	Coefficient estimate	*p*-value
Medicaid expansion (explanatory model)		
Non-Medicaid expansion states	ref	
Medicaid expansion states	0.0713 (−0.0076, 0.1503)	0.0764
Medicaid expansion*time (base model)		
Non-Medicaid expansion states*time	ref	
Medicaid expansion states*time	−0.0059 (−0.0225, 0.0107)	0.4856
Sociodemographic characteristics		
Non-Medicaid expansion states*time	ref	
Medicaid expansion states*time	−0.0063 (−0.0221, 0.0094)	0.4293
Minorities	−0.0042 (−0.0160, 0.0077)	0.4887
Bachelor’s degree	0.0067 (−0.0796, 0.0930)	0.8782
Unemployment	0.1068 (0.0313, 0.1824)	*0.0056*
Median household income	−0.0000 (−0.0000, 0.0000)	0.3813
Health delivery system characteristics		
Non-Medicaid expansion states*time	ref	
Medicaid expansion states*time	0.0237 (0.0067, 0.0406)	*0.0061*
Medicaid-to-Medicare fee index	0.9501 (0.4733, 1.4269)	*< 0.0001*
Medicaid spending per enrollee	−0.0003 (−0.0004, −0.0002)	*< 0.0001*
Medicaid income eligibility	−0.0046 (−0.0055, −0.0036)	* < 0.0001*
Health policy characteristics		
Non-Medicaid expansion states*time	ref	
Medicaid expansion states*time	−0.0042 (−0.0211, 0.0127)	0.6273
Republican state legislature	ref	
Democratic state legislature	0.1711 (0.0273, 0.3150)	*0.0197*
Republican state governor	ref	
Democratic state governor	0.1013 (−0.0179, 0.2205)	0.0957

Italic values are significant values

When we controlled for healthcare delivery characteristics, states that expanded their Medicaid programme saw a significant increase in the change in PH rates (coefficient estimate 0.0237, CI 0.0067, 0.0406, *p* = 0.0061). Medicaid spending per enrollee and Medicaid income eligibility were associated with significant decreases in rates of PH (improved access to care) (Table 3).

States that expanded Medicaid had lower rates of PH when controlling for state policy factors, but this difference was not significant. States where Democrats had control of the state legislature saw a significant increase in rates of PH (coefficient estimate 0.1711, CI 0.0273, 0.3150, *p* = 0.0197) (Table 3).

## Discussion

Our findings illustrate a slight decrease in the rate of PH over time for states that expanded Medicaid. Although not significant, our study shows that Medicaid expansion may have had a positive impact on primary care access. The most important aspect of this study was the health delivery system characteristics and their impact on PH rates across the states.

Our findings are similar to previous studies evaluating Medicaid expansion and its impact on access to primary care. In Oregon, after Medicaid expansion in 1994, researchers reported that annual PH rates increased from 46.1 to 54.9 per 10,000 persons [25]. In adjusted analysis, they reported that individuals had a higher likelihood of experiencing a PH (OR = 1.18, *p* < 0.001) [25]. An increase in PH rates should not signal policy failure but may hint at the need for more or better primary care services [25]. In another study in Massachusetts, researchers found that emergency department (ED) utilization for ACS conditions increased by 6.7 percentage points after healthcare reform (21.1% to 27.8%, *p* < 0.05) [26]. The authors note that access to primary care depends on primary care physician availability and convenience of emergency rooms; further, it may take time to see patients change care-seeking behaviours, which could also impact rates of PH [26]. Although Medicaid expansion may decrease the number of uninsured, it appears that after policy reform, poor access to care is still an issue, which may be for various reasons, including low Medicaid reimbursement, pent-up healthcare demand, ED convenience and hours, and illness severity.

### State health delivery system characteristics

Several state-level health delivery system characteristics (e.g., Medicaid income eligibility and Medicaid dollars spent per enrollee), which are derivative of healthcare policy, appear to be significantly associated with decreases in the rate of PH (improved access to care). The increase in Medicaid income eligibility and what states paid per Medicaid enrollee may have allowed more people to gain health insurance coverage, which may have increased access to care and led to a significant decrease in rates of PH.

In our study, the Medicaid-to-Medicare fee index appeared to be associated with higher rates of PH or worse access to primary care. This may be due to other factors that were not measured in the study. For example, if physicians are reimbursed at a higher rate for primary care services under Medicaid, ideally, we assume that more physicians would accept and treat more patients with Medicaid insurance, which could improve access to care and decrease rates of PH. However, this may not be the case at all. Researchers cite other factors like an increase in healthcare demand coupled with a declining number of providers who accept Medicaid that could impact access to care [27]. If there is a decrease in the number of providers who accept Medicaid, it could be due to a state’s low reimbursement rates for Medicaid primary care services. For example, for every $1.00 that Medicare pays for primary care services, Medicaid pays $0.48 for primary care services in New York (Medicaid expansion state) compared to $0.59 in the United States or $0.48 in Florida (non-Medicaid expansion state) [19].

Another factor that may contribute to our findings is the number of primary care physicians or medically underserved areas in states we examined. The availability of physicians could impact access to care. In one study, researchers found that as the number of family practitioners (FP) and general practitioners (GP) increased, the rate of avoidable hospitalizations decreased [28]. Researchers reported that for every increase in either a FP or GP, there was a 2.75 reduction in PH per 10,000 people [28]. Thus, it is plausible that the number of physicians (or lack thereof) could contribute to more PH, which is an indicator of poor primary care access.

### State sociodemographic characteristics

Our study shows that as the percentage of unemployment increases, so does the rate of PH. This may be due to other socioeconomic factors, including education, income, and even stress, which could impact health status. Individuals who are unemployed, lack monetary resources, are stressed about finances, and do not have health insurance may delay care and be hospitalized for preventable conditions. Several studies in the access-to-care literature have reported on delaying care due to costs [27, 29–31].

### State health policy characteristics

State legislatures controlled by Democrats saw a significant increase in rates of PH over time compared to Republican-controlled state legislatures. This could be due to the nature of Republican lawmakers, who promote more fiscally conservative policies that could impact health delivery system characteristics, leading to lower Medicaid income eligibility limits and the amount of money spent per Medicaid enrollee.

### Individual-level characteristics

A health insurance card does not lead to immediate access to healthcare [32]. An insurance card does not create convenient office hours or guarantee transportation to a medical provider, and does not address the unique needs of each patient [32]. In the Framework for the Study of Access [5], lack of transportation and special needs of the individual would be categorized as “characteristics of population at risk”, and convenient office house would fall under “consumer satisfaction”. In this same framework, all variables under “consumer satisfaction”, such as convenience, costs, quality, and courtesy, were not measured in this study and can certainly impact an individual’s access to primary care.

Another factor at the individual level may be that, although individuals gained a health insurance card, they do not necessarily know how to access primary care or have a trusting relationship with a provider they feel comfortable confiding in with their personal health information. According to the Agency for Healthcare Research and Quality [33], communication and trust are essential components in promoting access to healthcare. The time needed to (a) have individuals familiarize themselves with primary care practices and (b) develop a trusting relationship with a primary care provider is quite an important factor, and this aspect in healthcare practice takes time, especially for those who may have never had health insurance or a primary care provider. As more people gain insurance under the ACA and find a usual source of care or primary care doctor, they may be less likely to have a PH. We may need more time to see individuals find and develop a relationship with a trustworthy primary care provider before seeing a significant decrease in the rates of PH.

### State Medicaid characteristics

A separate factor at the state level was the approach to how Medicaid expansion states marketed Medicaid insurance to eligible populations, which was an unobserved factor in our research study. Medicaid expansion states may have varied by leadership, collaboration efforts, and other factors [34]. Medicaid expansion states may have had different strategies to target underserved groups or provide enrolment assistance like bilingual staff and interpretation services [34]. It is plausible that these unobserved characteristics in Medicaid expansion states could impact the rates of PH. If Medicaid expansion states were slow to market Medicaid or did not target traditionally underserved populations (e.g., minorities, uninsured, low-income groups), individuals may have missed the opportunity to get health insurance and endured poor access to primary care, which could lead to an increase in rates of PH in Medicaid expansion states.

## Limitations

Our study had several limitations, including possible administrative errors with ICD-9 codes. There was also an inability to measure unobserved differences in populations across the different states, including the differences in Medicaid enrolment and marketing strategies and patient care-seeking behaviours. Further, the analytical approach used may not capture nonlinear relationships between variables. Selection bias may be an issue, as authors utilized hospitalization data available at the time of the study. There are various methods to measure access to care, including using self-report or survey data and data from medically underserved areas. Lastly, findings are limited to the population studied.

## Conclusion

It is important to continue examining the impact of Medicaid expansion on access to primary care based on our findings regarding the health delivery system characteristics, a product of health policy reform. While the ACA Medicaid expansion happened approximately 10 years ago, the hospital administrative data to measure access to primary care after Medicaid expansion are still relatively new, including the transition from ICD-9 codes to ICD-10 codes. Research examining the effects of ACA Medicaid expansion is still in its early stages. As more administrative data become available, researchers should continue analysing more state hospital data over longer periods of time to see whether there is a significant effect on primary care access. Lastly, researchers should translate these findings into cost analysis for state policy-makers to make better-informed decisions for their constituents.

## Supplementary Information

**Additional file 1:** List of Ambulatory Care Sensitive (ACS) conditions and ICD-9 codes used to define preventable hospitalizations.

## Data Availability

The data that support the findings of this study are available from the Agency for Healthcare Research and Quality, but restrictions apply to the availability of these data, which were used under license for the current study, and so are not publicly available.
