# Correction to: Measuring the impact of the Afordable Care Act Medicaid expansion on access to primary care using an interrupted time series approach

**DOI:** 10.1186/s12961-021-00740-y

**Published:** 2021-06-01

**Authors:** Elizabeth A. Brown, Brandi M. White, Walter J. Jones, Mulugeta Gebregziabher, Kit N. Simpson

**Affiliations:** 1 Department of Clinical Sciences, College of Health Professions, Medical University of South Carolina, 151-B Rutledge Avenue, MSC 962, Charleston, SC 29425 USA; 2 Division of Health Sciences, Education, and Research, College of Health Sciences, University of Kentucky, Room 209C Wethington Building, 900 South Limestone Street, Lexington, KY 40536-0200 USA; 3 Department of Healthcare Leadership and Management, College of Health Professions, Medical University of South Carolina, 151-B Rutledge Ave, MSC 962, Charleston, SC 29425 USA; 4 Department of Public Health Sciences, College of Medicine, Medical University of South Carolina, 135 Cannon Street, Charleston, SC 29425 USA

## Correction to: Health Res Policy Sys (2021) 19:77 http://doi.org/10.1186/s12961-021–00730-0

It was highlighted that the original article [1] contained some typesetting errors in Table 1. Namely onn the row for "Medicaid $ per enrollee," there is an incorrect value (5,1205,120). That value should be 5,120 and in the Republican row there was an extra mention of value "34 (35.4)". This Correction article shows the correct table. The original article has been updated.

**Table 1:** Demographic variables and primary care access by Affordable Care Act Medicaid expansion status, 2012–2015

Demographic Variables^a^	NME^b^ States	ME^c^ States	p-value
Access to Care (n, %)			
Preventable Hospitalizations	1,096,631 (52.1)	1,006,483 (47.9)	0.2670
State Population	6,127,025	6,191,002	0.8776
18–64 years old			
Race (%)			
Minorities	41.7	38.9	0.9457
Education (%)			
High School Graduate	87.3	86.1	0.0676
Bachelor’s Degree	27.4	30.2	**0.0194**
Income ($)			
MHI	49,006	56,240	0.0005
Per Capita Income	26,381	29,865	0.0194
Poverty (%)			
Below FPL	15.3	15.6	0.1704
Income < 10 k	7.2	7.6	0.2244
Food Stamps	14.0	13.5	0.2546
Unemployment	7.0	7.2	0.2595
Health care delivery characteristics			
Physician fee index ($)^d^	0.60	0.58	0.8029
Medicaid $ per enrollee ($)^e^	3,336	5,120	** < 0.0001**
Income Eligibility, FPL (%)^f^Income Eligibility, FPL (%)^f^	80.6	124.1	** < 0.0001**
Governor Political Affiliation			
Democratic	0 (0.0)	27 (100.0)	** < 0.0001**
Republican	62 (64.6)	34 (35.4)	
Control of State Legislature			
Democratic	0 (0.0)	45 (100.0)	** < 0.0001**
Republican	62 (79.5)	16 (20.5)	

^a^T-test for continuous variables and chi-square test for categorical variables

^b^NME: Non-Medicaid expansion states include South Carolina, Wisconsin, Georgia, and Florida

^c^ME: Medicaid expansion states include Arizona, Kentucky, New Jersey, and New York

^d^Medicaid-to-Medicare Fee Index

^e^Medicaid spending per enrollee

^f^Average of pre- and post- ACA FPL Medicaid Income Eligibility Limits for parents of dependent children (in a family of three)

## References

[1] Brown EA, White BM, Jones WJ, Gebregziabher M, Simpson KN (2021). Measuring the impact of the Afordable Care Act Medicaid expansion on access to primary care using an interrupted time series approach. Health Res Policy Sys.

