# Supplementary material for: Measuring the impact of the Affordable Care Act Medicaid expansion on access to primary care using an interrupted time series approach
Source: Health Res Policy Syst. 2021 May 6;19:77. doi: 10.1186/s12961-021-00730-0 (PMC8101185; doi:10.1186/s12961-021-00730-0)
Supplement: Supplementary file 1 — Additional file 1: List of Ambulatory Care Sensitive (ACS) conditions and ICD-9 codes used to define preventable hospitalizations. [file 12961_2021_730_MOESM1_ESM.doc]

**Supplement 1_online_supp: List of Ambulatory Care Sensitive (ACS) conditions and ICD-9 codes used to define preventable hospitalizations**

| **Ambulatory Care Sensitive (ACS) Conditions** | **References**  **[ICD-9 Codes]** | **Comments** |
| --- | --- | --- |
| 1. Angina pectoris | Brown, Goldacre, Hicks, Rourke, McMurtry, Brown, & Anderson^1^  [413]  Millman^2^  [411.1, 411.8, 413]  Sanderson & Dixon^3^  [413] | - Exclude cases with a surgical procedure [01-86.99]^2^ - Intermediate coronary syndrome (411.1) - Other acute and subacute forms of ischemic heart disease (411.8) - Angina pectoris (413) |
| 1. Asthma | Brown, Goldacre, Hicks, Rourke, McMurtry, Brown, & Anderson^1^  [493]  Millman^2^  [493]  Sanderson & Dixon^3^  [493.0, 493.9] | - Asthma (493) - Extrinsic asthma (493.0) - Asthma unspecified (493.9) |
| 1. Bacterial Pneumonia | Millman^2^  [481, 482.2, 482.3, 482.9, 483, 485, 486]  Sanderson & Dixon^3^  [481, 485, 486] | - Exclude cases with secondary diagnosis of sickle cell [282.6] and patients < 2 months^2^ - Pneumococcal pneumonia (481) - Pneumonia due to Hemophilus influenza (482.2) - Pneumonia due to streptococcus (482.3) - Bacterial pneumonia, unspecified (482.9) - Pneumonia due to other specified organism (483) - Bronchopneumonia, organism unspecified (485) - Pneumonia, organism unspecified (486) |
| 1. Cellulitis | Millman^2^  [681, 682, 683, 686]  Sanderson & Dixon^3^  [682.0] | - Exclude cases with surgical procedure [01-86.99], except skin incision and subcutaneous tissue [86.0] where it is just listed as surgical procedure^2^ - Cellulitis and abscess of finger and toe (681) - Other cellulitis and abscess (682) - Cellulitis and abscess of face (682.0) - Acute lymphadenitis (683) - Other local infections of skin and subcutaneous tissue (686) |
| 1. Chronic Obstructive Pulmonary Disease (COPD) | Millman^2^  [491, 492, 494, 496, 466.0]  Sanderson & Dixon^3^  [496, 466.1] | - Acute bronchitis [466.0] only with secondary diagnosis of 491, 492, 494, 496^2^ - Chronic bronchitis (491) - Emphysema (492) - Bronchiectasis (494) - Chronic airway obstruction (496) - Acute bronchitis (466.0) - Acute bronchiolitis (466.1) |
| 1. Congestive Heart Failure (CHF) | Brown, Goldacre, Hicks, Rourke, McMurtry, Brown, & Anderson^1^  [428]  Millman^2^  [428, 402.01, 402.11, 402.91, 518.4]  Sanderson & Dixon^3^  [428.0, 428.1] | - Exclude cases with surgical procedures [36.01, 36.02, 36.05, 36.1, 37.5, or 37.7]^2^ - Congestive Heart Failure (428) - Malignant Hypertensive Heart Disease with Heart Failure (402.01) - Benign Hypertensive Heart Disease with Heart Failure (402.11) - Unspecified Hypertensive Heart Disease with Heart Failure (402.91) - Congestive Heart Failure, unspecified (428.0) - Left heart failure (428.1) - Acute edema of lung, unspecified (518.4) |
| 1. Convulsions “B” | Millman^2^  [780.3]  Sanderson & Dixon^3^  [780.3] | - Use for those age > 5^2^ - Convulsions (780.3) |
| 1. Dehydration - volume depletion | Millman^2^  [276.5] | - Examine principal and secondary diagnoses separately^2^ - Volume depletion disorder (276.5) |
| 1. Diabetes “A” | Millman^2^  [250.1, 250.2, 250.3]  Sanderson & Dixon^3^  [250.1] | - Diabetes with ketoacidosis (250.1) - Diabetes with hyperosmolarity (250.2) - Diabetes with other coma (250.3) |
| 1. Diabetes “B” | Millman^2^  [250.8, 250.9]  Sanderson & Dixon^3^  [250.4] | - Diabetes with other specified manifestations (250.8) - Diabetes with unspecified complication (250.9) - Diabetes with renal manifestations (250.4) |
| 1. Diabetes “C” | Millman^2^  [250.0]  Sanderson & Dixon^3^  [250.0] | - Diabetes mellitus without mention of complication (250.0) |
| 1. Gastroenteritis | Millman^2^  [558.9]  Sanderson & Dixon^3^  [558] | - Other and unspecified noninfectious gastroenteritis and colitis (558.9) (558) |
| 1. Gastrointestinal ulcer | Brown, Goldacre, Hicks, Rourke, McMurtry, Brown, & Anderson^1^  [531, 532, 533, 534]  Sanderson & Dixon^3^  [531.9, 532.9, 532.7] | - Gastric ulcer (531) - Gastric ulcer, unspecified (531.9) - Duodenal ulcer (532) - Chronic duodenal ulcer (532.7) - Duodenal ulcer, unspecified (532.9) - Peptic ulcer (533) - Gastrojejunal ulcer (534) |
| 1. Grand mal status and other epileptic convulsions | Millman^2^  [345]  Sanderson & Dixon^3^  [345.1, 345.9] | - Epilepsy and recurrent seizures (345) - Generalized convulsive epilepsy (345.1) - Epilepsy, unspecified (345.9) |
| 1. Hypertension / Malignant Hypertension | Brown, Goldacre, Hicks, Rourke, McMurtry, Brown, & Anderson^1^  [401.0, 402.0, 403.0, 404.0]  Millman^2^  [401.0, 401.9, 402.00, 402.10, 402.90] | - Exclude cases with procedures [36.01, 36.02, 36.05, 36.1, 37.5, 37.7]^2^ - Malignant hypertension (401.0) - Unspecified hypertension (401.9) - Malignant hypertensive heart disease (402.0) - Malignant hypertensive heart disease without heart failure (402.00) - Benign hypertensive heart disease without heart failure (402.10) - Unspecified hypertensive heart disease without heart failure (402.90) - Malignant hypertensive renal disease (403.0) - Hypertensive heart and chronic kidney disease (404.0) |
| 1. Hypoglycemia | Millman^2^  [251.2]  Sanderson & Dixon^3^  [251.2] | - Hypoglycemia (251.2) |
| 1. Kidney/Urinary Tract Infection | Millman^2^  [590, 599.0, 599.9] | - Infections of kidney (590) - Urinary tract infection, site not specified (599.0) - Unspecified disorder of urethra and urinary tract (599.9) |
| 1. Severe ENT (ears, nose, and/or throat) infections | Brown, Goldacre, Hicks, Rourke, McMurtry, Brown, & Anderson^1^  [382]  Millman^2^  [382, 462 463, 465, 472.1]  Sanderson & Dixon^3^  [465.9] | - Exclude cases with otitis media [382] and myringotomy with insertion tube [20.01]^2^ - Otitis media (382) - Acute pharyngitis (462) - Acute tonsillitis (463) - Acute upper respiratory infections (465) - Acute upper respiratory infections, unspecified (465.9) - Chronic pharyngitis (472.1) |

References

1. Brown, A. D., Goldacre, M. J., Hicks, N., Rourke, J. T., McMurtry, R. Y., Brown, J. D., & Anderson, G. M. (2001). Hospitalization for ambulatory care-sensitive conditions: a method for comparative access and quality studies using routinely collected statistics. *Can J Public Health, 92*(2), 155-159.
2. Millman, M. (1993). *Access to Health Care in America*. Washington, D.C.: National Academy Press.
3. Sanderson, C., & Dixon, J. (2000). Conditions for which onset or hospital admission is potentially preventable by timely and effective ambulatory care. *J Health Serv Res Policy, 5*(4), 222-230.
